# Fitting a Hearing Aid on the Better Ear, Worse Ear, or Both: Associations of Hearing-aid Fitting Laterality with Outcomes in a Large Sample of US Veterans

**DOI:** 10.1177/23312165231195987

**Published:** 2023-08-24

**Authors:** Oliver Zobay, Graham Naylor, Gabrielle H. Saunders, Lauren K. Dillard

**Affiliations:** 1 Hearing Sciences, Mental Health and Clinical Neurosciences, School of Medicine, 6123 University of Nottingham, Nottingham, UK; 2 VA Rehabilitation R&D, National Center for Rehabilitative Auditory Research, Portland, OR, USA; 3 574111 NIHR Nottingham Biomedical Research Centre, Nottingham, UK; 4 Manchester Centre for Audiology and Deafness, School of Health Sciences, The University of Manchester, Manchester, UK; 5 Department of Otolaryngology–Head & Neck Surgery, Medical University of South Carolina, Charleston, SC, USA

**Keywords:** hearing aids, hearing-aid fitting, hearing asymmetry, unilateral hearing loss

## Abstract

Longitudinal electronic health records from a large sample of new hearing-aid (HA) recipients in the US Veterans Affairs healthcare system were used to evaluate associations of fitting laterality with long-term HA use persistence as measured by battery order records, as well as with short-term HA use and satisfaction as assessed using the International Outcome Inventory for Hearing Aids (IOI-HA), completed within 180 days of HA fitting. The large size of our dataset allowed us to address two aspects of fitting laterality that have not received much attention, namely the degree of hearing asymmetry and the question of which ear to fit if fitting unilaterally. The key findings were that long-term HA use persistence was considerably lower for unilateral fittings for symmetric hearing loss (HL) and for unilateral worse-ear fittings for asymmetric HL, as compared to bilateral and unilateral better-ear fittings. In contrast, no differences across laterality categories were observed for short-term self-reported HA usage. Total IOI-HA score was poorer for unilateral fittings of symmetric HL and for unilateral better-ear fittings compared to bilateral for asymmetric HL. We thus conclude that bilateral fittings yield the best short- and long-term outcomes, and while unilateral and bilateral fittings can result in similar outcomes on some measures, we did not identify any HL configuration for which a bilateral fitting would lead to poorer outcomes. However, if a single HA is to be fitted, then our results indicate that a better-ear fitting has a higher probability of long-term HA use persistence than a worse-ear fitting.

## Introduction

Several factors, including patient preferences, patient pathologies, and potential outcomes, influence the decision as to whether to fit one or two hearing aids (HAs), and if the former, which ear should be fitted. To navigate this decision process, patients and care providers should have evidence-based information and recommendations available because there remains a debate as to whether bilateral or unilateral fittings lead to better outcomes. Indeed, a recent Cochrane review (Schilder et al., 2017) concluded that the evidence available from randomized controlled trials (RCTs) is of very low quality. Given the lack of high-quality RCTs, it is of value to investigate what evidence other sources of data can provide.

An analysis of a Swedish database containing more than 100,000 completed International Outcome Inventory for Hearing Aids (IOI-HA; Cox et al., 2000) questionnaires showed that people with a bilateral HA fitting had significantly higher scores on all seven IOI-HA items compared to those with a unilateral fitting (Arlinger et al., 2017). Likewise, data from about 132,000 HA users from Germany, France, and the United Kingdom found better outcomes for bilateral users than unilateral users in terms of satisfaction with the features and performance of the HAs, and daily HA use (Bisgaard & Ruf, 2017).

Similarly, several smaller-scale studies also showed better outcomes with bilateral than unilateral fittings. Specifically, in a prospective study of 214 participants, Boymans and Dreschler (2011) found bilateral fittings superior in terms of the users’ localization ability and speech understanding and concluded that “the benefit of a second HA is obvious” (p. 294). They recommended that “every hearing-impaired subject should start with a bilateral fitting to experience the benefits and the drawbacks” (p. 294). Likewise, van Schoonhoven et al. (2016) reported a bilateral benefit for listening effort, speech reception in noise and localization among a group of 21 participants with moderate-to-severe hearing loss (HL) as well as for listening effort for 19 participants with mild HL, with there being no significant disadvantage of a second HA in any of their laboratory-based testing. In a third study, Noble and Gatehouse (2006) used the Speech, Spatial, and Qualities (SSQ) self-report scale (Gatehouse & Noble, 2004) to compare three audiometrically matched patient groups (no HA *N* = 63, unilateral fitting *N* = 69, bilateral fitting *N* = 34). It was found that participants with bilateral fittings had better SSQ scores than those with unilateral fittings for hearing speech in demanding contexts, spatial hearing, and listening effort. A similar result was reported in a study by Most et al. (2012) which found that bilateral HA users (*N* = 46) had better SSQ scores than unilateral users (*N* = 34) on the speech and spatial scales. More specifically, for the subgroup with symmetric unaided HL (*N* = 34 bilateral; 26 unilateral), bilateral users showed significantly better outcomes on all three scales of the SSQ while for asymmetric HL (*N* = 12/8), no significant intergroup differences were found.

Even though these results demonstrate that bilateral fittings are generally advantageous, a considerable number of patients nonetheless receive a unilateral HA fitting, with recent studies reporting proportions between about 20% and 40% (Arlinger et al., 2017; Boymans et al., 2009). Reasons given for this choice include cost, age (with older individuals performing better in noise with a unilateral fitting), binaural interference, decreased occlusion, and the reduced effort of handling a single device (Henkin et al., 2007; Jespersen et al., 2006; Schilder et al., 2017; Walden & Walden, 2005). Furthermore, unilateral fittings are sometimes considered advantageous when it comes to the use of telephones or when the capacity of the unaided ear is “relatively good […] or just worse” (Boymans & Dreschler, 2011, p. 286).

The relatively high proportion of patients still receiving unilateral fittings shows that the debate about HA laterality remains of considerable clinical significance. The current work aims to contribute to this discussion by addressing two issues—(1) how HA use and outcomes are affected by the combination of hearing asymmetry and severity and (2) selection of which ear to fit if fitting unilaterally.

There is little published regarding the degree of asymmetry and severity. Most studies include participants with limited hearing asymmetry, or the degree of asymmetry is not examined. Specifically, Boymans et al. (2009) and van Schoonhoven et al. (2016) restricted their comparisons to patients with symmetric HL while the audiometric matching procedure of Noble and Gatehouse (2006) resulted in the removal of patients at both ends of the HL spectrum. Arlinger et al. (2017) and Bisgaard and Ruf (2017) did not control for HL in their analyses. In other words, there is a need to examine the relationship between the degree of hearing asymmetry and the outcome of a unilateral HA fitting. It seems plausible that a unilateral fitting could be particularly appropriate for highly asymmetrical losses, especially for individuals with a profound HL in their worse ear (WE) and/or individuals with normal hearing or very mild HL in their better ear (BE). In the former case, fitting the WE will likely be of limited benefit, whereas in the latter case, there is little necessity to fit the BE.

The question of whether to fit the BE or the WE in a unilateral fitting was investigated in a series of papers by Swan and colleagues. Swan et al. (1986, 1987) described the results of a cross-over trial with new HA users in which a unilateral HA was sequentially fitted to both ears. In terms of preference, of the 23 patients with asymmetric HL, 13 reported a laterality preference due to hearing ability which invariably was for the WE. None of the 23 patients claimed to hear better with a better-ear fitting. In terms of performance, more benefit was obtained for a *diotic* listening task when the aid was in the BE, while for a *dichotic* task performance was better when the WE was aided (Swan & Gatehouse, 1987a, 1987b). The authors interpreted these results as supporting the hypothesis that patients’ choice of laterality is influenced by a desire to minimize disability in the most disadvantageous listening situations.

Other authors also give recommendations on choosing laterality for unilateral fitting but do this without providing empirical evidence. As a simple rule, Dillon (2012, p. 462) suggests fitting the ear that has four-frequency average closer to 60 dB but also lists several non-audiometric factors that should be taken into consideration such as dexterity or medical complications in one of the ear canals. Kiessling et al. (2006) describe a criterion based on speech intelligibility and discrimination in the better and worse ear.

A major obstacle to studying associations between laterality of HA fittings, hearing asymmetry and HA outcomes is the availability of data with sample sizes that provide appropriate statistical power. We are fortunate to have access to a large clinical dataset arising from electronic health records (EHRs) collected by the US Veterans Health Administration (VHA) (see Dillard et al., 2020; Saunders et al., 2021; Zobay et al., 2021 for details). This large dataset provides us with ecologically valid data that allow us to draw conclusions about the implications of clinical practices on outcomes and thus make recommendations for future clinical practice. In the present paper, we leverage our dataset to answer the following questions: (1) *what is the association between the laterality of a HA fitting and long-term HA use persistence*, (2) *what are the associations between the laterality of a HA fitting and short-term HA usage and reported HA outcome assessed using the IOI-HA?*

Our dataset allows us to separately—and with relatively fine granularity–analyze data for individuals with various degrees of hearing asymmetry, and to examine how this interacts with outcomes for bilateral and unilateral HA fittings and ear that is fitted. The aim of our statistical analyses is to compare outcomes between bilateral fittings and unilateral better-ear and worse-ear fittings for different degrees of hearing asymmetry, with a view to making clinical recommendations.

## Materials and Methods

This work was approved by the Institutional Review Board and the Research and Development Committee of the VA Portland Health Care System (Study #03566), Data Access Request Tracker (tracking number 2014-11-066-D-A04), and VA Patient Care Services.

### Dataset

Our initial dataset consisted of EHRs for 731,213 patients for whom HAs were ordered through U.S. Department of Veterans Affairs (VA) audiology between April 2012 and October 2014. For all these patients, the dataset included demographic information, diagnostic (International Classification of Diseases [ICD]-9/10) and procedural (current procedural terminology [CPT]) codes related to health care provision in the VA system between January 2007 and December 2017, as well as records of their HA orders between April 2012 to October 2014 and of all HA battery orders placed through the VA health system between April 2012 and December 2017. Within the dataset, 570,295 patients had full audiometric data (i.e., at least one audiogram with left and right thresholds at 500; 1,000; 2,000; and 4,000 Hz taken between April 2012 and October 2014), and 146,699 patients had IOI-HA responses submitted between 14 and 180 days after their HA fitting. A full description of the dataset and the pre-processing conducted can be found in Dillard et al. (2020) and Saunders et al. (2021).

### Patient Sample

The analyses reported in this paper used different subsets of the full dataset, but in all cases, the following initial filters were applied.

- We limited our analyses to new HA recipients, i.e. patients flagged as having had no previous HA orders in the VA system, in order to increase comparability between users and also to avoid the possibility that what appeared to be a unilateral fitting was in fact a replacement of one HA in a bilateral fitting.
- We only included patients aged 50 years and over to make the sample more homogeneous and to focus on age-related HL.
- We included only patients for whom full audiometric data were available and who had a HA fitting within 180 days of their HA order (see Saunders et al. (2021) on how fittings were identified in the absence of a direct marker in the dataset).
- We included only individuals who had a single HA order within the time period of interest. Individuals with more than one HA order were excluded to avoid confounding and complications with the analysis. In some instances, patients with multiple orders could be identified unambiguously from the HA order records which were available in our dataset until October 31, 2014. In other cases, patients were excluded because it seemed likely from certain CPT codes in their EHRs that they had another fitting after the cut-off. The codes (V5011—fitting/orientation/checking of HA; V5020—conformity evaluation) were selected based on their close correlation with HA orders before 31 October 2014.
- We excluded all patients with codes related to cochlear implants (CPT codes 69930, 92601-92604, L8614-L8629).

### HL Patterns

We computed left and right four-frequency pure-tone averages (PTAs) from thresholds at 500; 1,000; 2,000; and 4,000 Hz (if a patient had multiple full audiograms these were averaged). The ear with the lower PTA was designated the BE. Asymmetric hearing was defined as an interaural PTA difference of more than 10 dB HL. We also identified patients with single-sided deafness (SSD), i.e. patients with one “dead” ear for which thresholds could not be measured.

**Figure 1.**
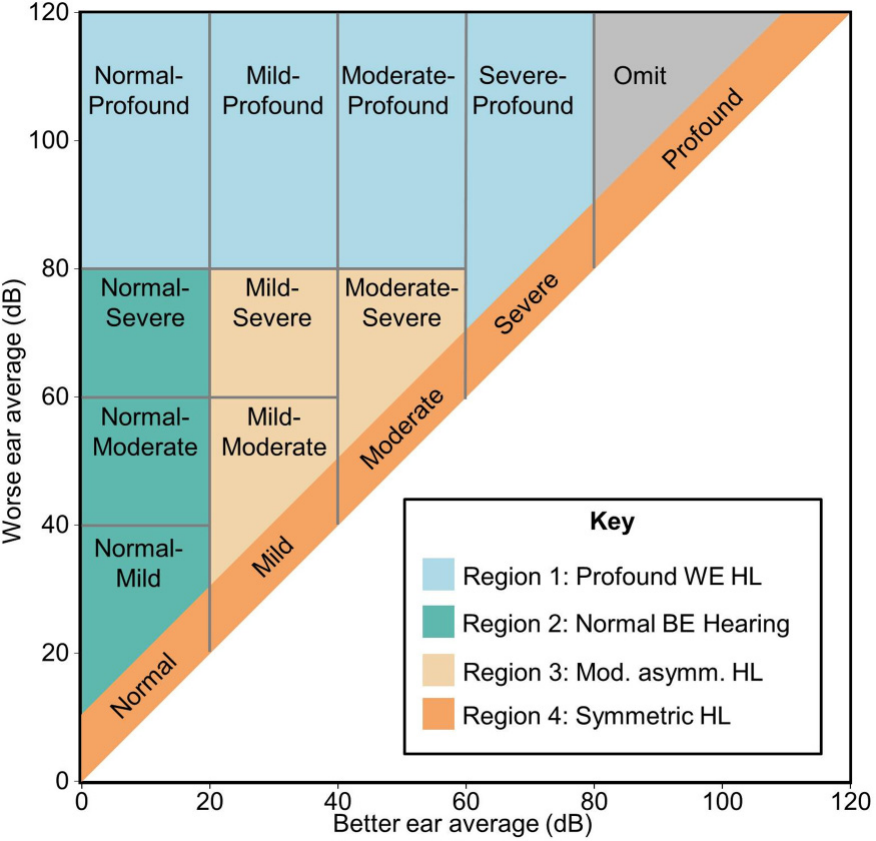
Classification of binaural hearing loss as a function of four-frequency better-ear and worse-ear PTA. Patients with binaural PTA differences of 10 dB or less are considered as having symmetric HL and are grouped according to the hearing level of the better ear into categories ranging from normal hearing to profound HL. Patients with asymmetric HL are grouped according to their binaural hearing levels as shown in the diagram. Patients with profound-profound asymmetric HL are omitted for simplicity. Background colors reflect the four main HL configurations (regions 1–4) defined in the main text. BE = better ear, WE = worse ear; PTA = pure-tone averages; HL = hearing loss.

HLs were classified into categories based on PTA in the BE and WE (see Figure 1). Hearing in the BE was categorized as normal (PTA ≤ 25 dB), mild loss (25 dB < PTA ≤ 40 dB), moderate loss (40 dB < PTA ≤ 60), severe loss (60 dB < PTA ≤ 80 dB), and profound loss (80 dB < PTA) (World Health Organization, 1991). Symmetric HL was then classified as normal, mild, etc. according to the better-ear PTA. Categories for asymmetric HL were constructed in a similar manner based on a corresponding discretization of the worse-ear HL.

From this classification, we defined four main regions of HL configurations, based on the difference between the worse-ear average (WEA) and better-ear average (BEA) PTA. These regions reflect the main scenarios for which we wanted to investigate the association of HA laterality with our outcomes of interest. Note that data from individuals with profound but asymmetric loss in both ears were excluded as the proportion of such patient numbers was very small (about 0.1%).

- Region 1 (blue in figures): Profound HL in the WE (WEA > 80 dB) with normal hearing to severe HL in the BE.
- Region 2 (turquoise in figures): Normal hearing in the BE (BEA ≤ 25 dB) and mild to severe HL in the WE (WEA ≤ 80 dB).
- Region 3 (orange in figures): Moderately asymmetric HL with BEA > 25 dB, WEA ≤ 80 dB.
- Region 4 (red in figures): Symmetric HL with a binaural PTA difference of at most 10 dB.

### Laterality of HA Fittings

The HA records indicated whether HAs had been ordered for both ears (bilateral fitting) or for just one ear (unilateral fitting). Based on the combination of HL region and HA fitting laterality five categories of HA fitting were defined, as follows:

1. Bilateral fitting that can occur in regions 1, 2, 3, and 4.
2. Unilateral better-ear fitting that can occur in regions 1, 2, and 3.
3. Unilateral worse-ear fitting that can occur in regions 1, 2, and 3.
4. Unilateral fitting in the presence of symmetric HL that can occur only in region 4.
5. Unilateral fitting in the presence of SSD on the unaided side that can occur only in region 1.

### Outcome Variables

*Long-term HA use persistence.* A measure of HA use persistence was computed based on the history of battery orders made by the patient. Specifically, a HA recipient is considered persistent at two years after their HA fitting if they had at least one battery order within the 18-month-period preceding the 2-year mark. Battery supplies are calibrated to 6-month full-time use of the provided HAs; in particular, a unilaterally fitted patient will receive half as many batteries as a bilateral patient. Our rule for assessing persistence is thus applicable in the same way to both bilaterally and unilaterally fitted patients. Furthermore, our definition implies that persistence corresponds to at least a third of full-time usage. A more detailed discussion of this measure is provided in the works of Saunders et al. (2021) and Zobay et al. (2021). In these articles, we show that higher HA use persistence as per our measure is positively associated with ongoing hearing care visits, more severe HL, and higher HA use reported on the IOI-HA. These and further reported findings provide evidence of the validity of our HA use persistence measure.

*Short-term HA usage and satisfaction.* Short-term usage was defined based on responses to item 1 of the IOI-HA questionnaire (“Think about how much you used your present hearing aid(s) over the past two weeks. On an average day, how many hours did you use the hearing aid(s)?”. Response options: None, less than 1 h, 1–4 h, 4–8 h, more than 8 h). Responses were dichotomized into two categories: < 4 h HA use/day and ≥4 h HA use/day. Four hours was selected as the cut point because it corresponds to at least a third of full-time usage if full-time use is considered to be 12 h/day. This definition is thus consistent with our metric of long-term HA use persistence. The total score on the IOI-HA was used as a measure of HA satisfaction (Cox & Alexander, 2002). The score ranges from 7 to 35 and was treated as a continuous variable.

### Statistical Analyses

All computational work was performed with R software, version 4.1.2 (R Core Team, 2021). We conducted three main analyses which considered long-term HA use persistence (Analysis 1), short-term HA usage (Analysis 2a) and total IOI-HA score (Analysis 2b). In the first step of each of these analyses, raw proportions or means together with patient counts were used to describe the dependence of the respective outcome measure on HA laterality and binaural HL patterns in the absence of any adjustments for other variables. In the second step, we investigated the connection between HA laterality and the outcome using logistic or linear regression models. Such regressions can be used to statistically model the association between a binary or continuous outcome variable, respectively, and the predictor variable(s) of interest after accounting for possible confounding effects from other covariates (Bland, 2015). The predictor variable of interest in this paper is HA laterality, and results are reported for its statistical main effect and for comparisons between the different types of fitting. The comparisons are presented in terms of adjusted odds ratios (ORs) or beta coefficients with corresponding 95% confidence intervals (95% CI) and *p*-values. Examples clarifying the interpretation of ORs and beta coefficients will be given in the “Results” section.

To account for the possibility that the statistical associations differed substantially in the four hearing regions, and to best quantify differences in bilateral and unilateral better-ear and worse-ear fittings, separate regressions were fitted for each region shown in Figure 1.

All regression models included HA laterality, the binaural HL pattern and selected covariates (see below) as main effects, i.e., Outcome ∼ Laterality + HL Pattern + Covariates in symbolic notation (R Core Team, 2021). The structure of the laterality factor varied from region to region; its categories included bilateral fittings for all regions, whereas unilateral fitting categories comprised better-ear fittings with and without SSD, respectively, in region 1 (profound worse-ear HL), as well as better-ear and worse-ear fittings, respectively, in regions 2 (normal better-ear hearing) and 3 (moderately asymmetric HL). In region 4 (symmetric HL), we cannot distinguish between worse and better-ear fittings so there is only a single unilateral category. Worse-ear fittings in region 1 were excluded due to small patient counts. The levels of the HL Pattern factor are given by the HL categories of the respective region as shown in Figure 1.

Our regression models included a range of covariates to account for possible confounding effects in the relationship between the outcome measures and HA laterality. To select the covariates, we extensively studied candidate variables derivable from our datasets for which a relationship with the outcomes might be expected. The final selection was based on subject-matter and statistical considerations, but we only retained variables with a statistically significant effect in the regression models. The selected covariates included variables related to demographics and hearing aids (i.e., HA type and short-term care after HA fitting) as well as a multimorbidity index. A detailed description of the variables can be found in the Supplemental Material. About 4% of patients had missing values for one or more covariates. To avoid losing the affected cases, these data were imputed using hot-deck imputation as implemented in the R package VIM version 6.2.2 (Kowarik & Templ, 2016). For reasons of conciseness, we do not report regression results for any covariates included in the models.

We also investigated statistical models including an interaction term between Laterality and HL Pattern, but this term was generally found not to be statistically significant or otherwise useful for any of the persistence and IOI-HA models. Lastly, we conducted several relevant sensitivity analyses that are presented in the “Discussion” section and in the Supplemental Material.

### Analysis 1: Association of HA Laterality with Long-Term HA use Persistence

The objective of analysis 1 was to compare long-term HA use persistence for patients with bilateral and unilateral HA fittings. For patients with asymmetric HL, we distinguished between unilateral better-ear and worse-ear fittings. Persistence was assessed for patients who had a valid HA fitting and who were still alive 2 years after the fitting.

### Analysis 2: Association of HA Laterality with Short-Term HA Usage and Total IOI-HA Score

The objective of analysis 2 was to evaluate the association of laterality with HA usage shortly after fitting as determined by self-report on the IOI-HA item 1, and with HA satisfaction as measured using the IOI-HA total score. For this analysis, we included only patients with a valid IOI-HA submission up to 180 days after HA fitting (Saunders et al., 2021). We also only included patients fitted before 1 May 2014. Since HA order data are available until October 31, 2014, this cut-off on the fitting date ensures that patients did not receive a second device before their IOI-HA submission.

## Results

### Descriptive Analyses: Patient Distributions by HA Laterality and HL Configuration

**Figure 2.**
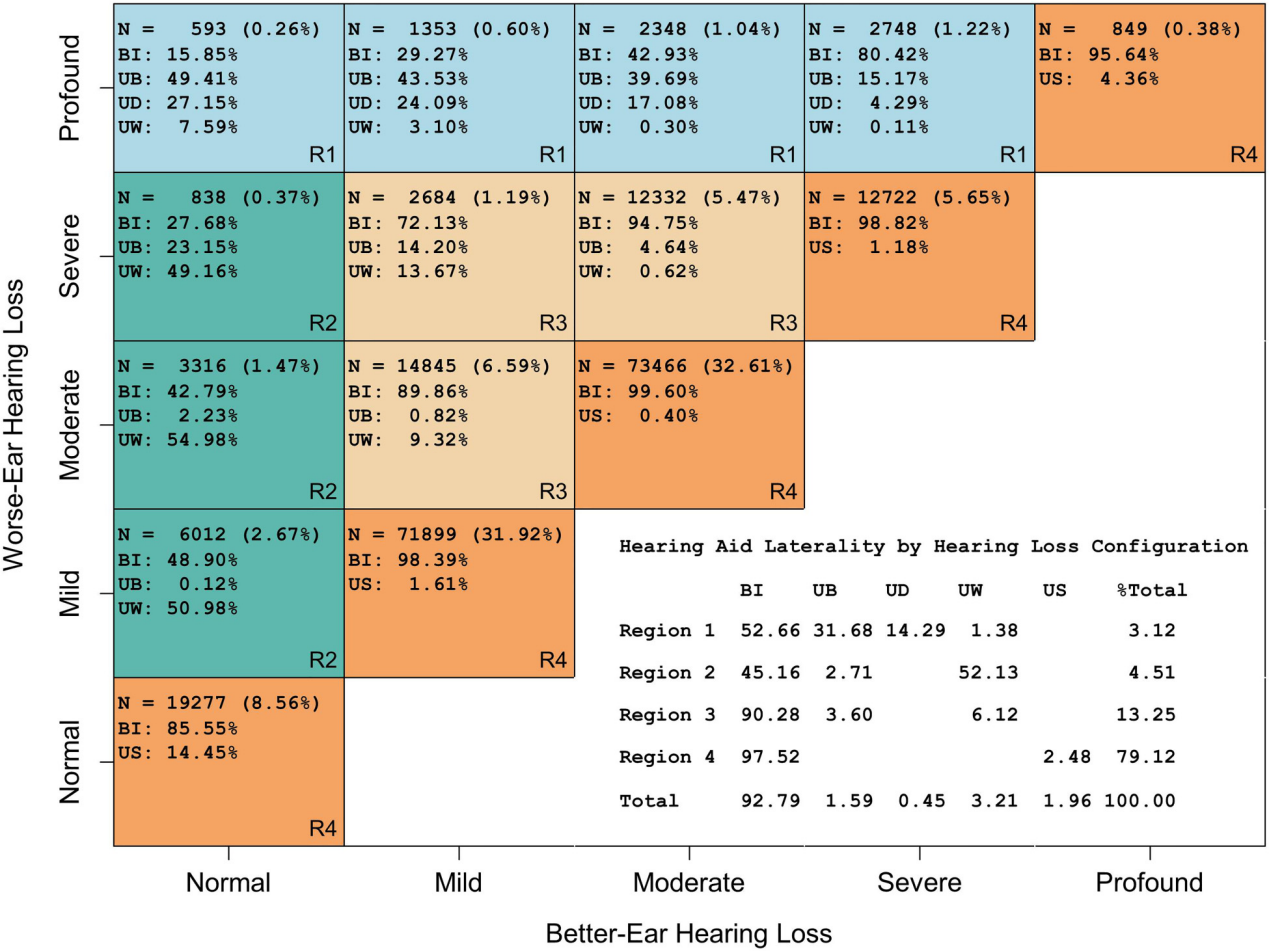
Distribution of HA fitting laterality by binaural HL configuration. First row in each cell shows the patient count (*N*) for the cell and proportion of overall patient number, subsequent rows show distribution (%) across laterality types within the cell. Counts include new patients with a single recorded HA order and fitting by 30 April 2014. Color scheme reflects the four main types of HL configuration (regions 1–4). The table in the bottom right corner provides distributions at the level of the four regions and for the full sample. BI = bilateral; UB/UW = unilateral better/worse ear; UD = unilateral without measurable PTA in contralateral ear, i.e., single-sided deafness; US = unilateral with symmetric HL; PTA = pure-tone averages; HL = hearing loss; HA = hearing aid.

Figure 2 shows the distribution of laterality of HA fitting as a function of patients’ binaural HL pattern for 215,879 patients (filtered from the full sample as described above with the additional requirement of a single recorded fitting by 30 April 2014 and no further HA orders before 1 November 2014). In total, 79.1% of patients had symmetric HL (region 4), 3.1% had profound HL in the WE (region 1), 4.5% had normal hearing in the BE (region 2) with the remaining 13.3% having moderately asymmetric HL (region 3). Altogether, 92.8% of patients were fitted bilaterally, 2.0% received a unilateral device in the presence of symmetric HL and 3.2% received a unilateral fitting for their WE. The proportions of better-ear fittings for patients without and with SSD are 1.6% and 0.5%, respectively.

### Analysis 1: Association Between HA Laterality and Long-Term HA use Persistence

**Figure 3.**
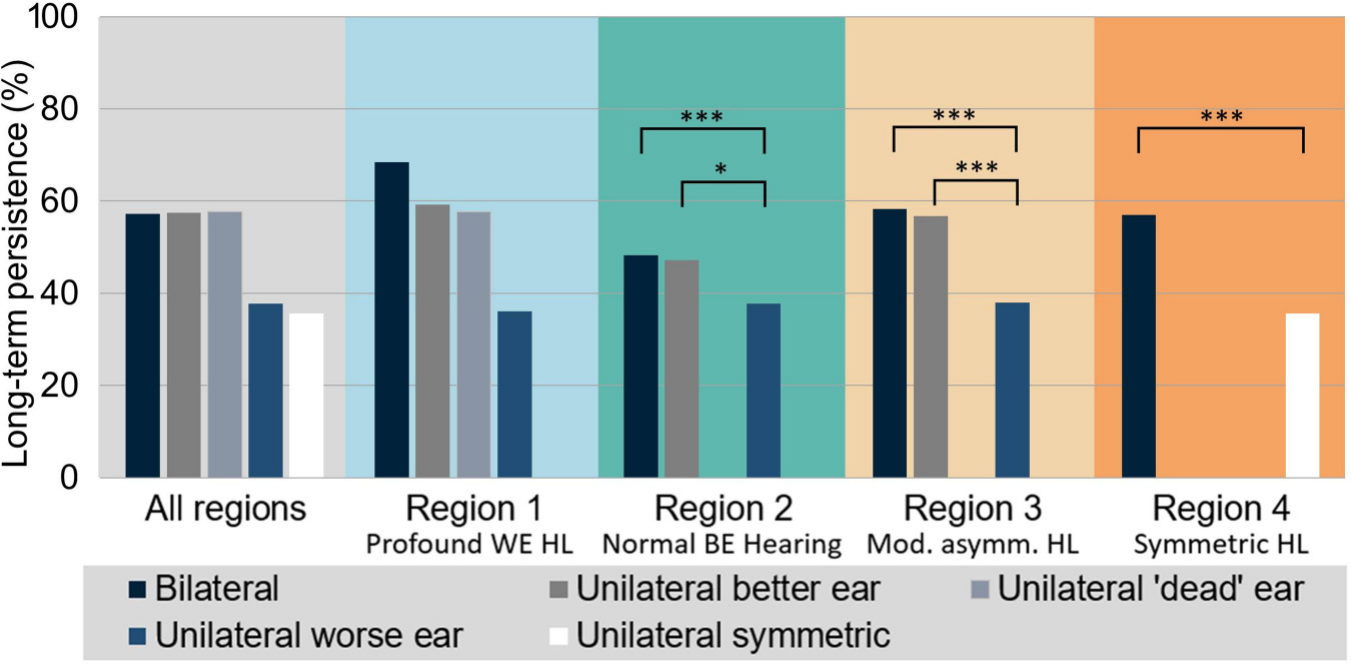
Long-term HA uses persistence for the different types of HA laterality across the whole sample and separately for regions 1–4. Statistically significant comparisons identified by the logistic regression models are highlighted (see Table 1). BE = better ear, WE = worse ear; HA = hearing aid; HL = hearing loss.

There were 249,719 patients included in analysis 1. Figure 3, Figure S1, and Table 1 summarize the results of descriptive and regression analyses that characterize the association between HA fitting laterality and long-term HA use persistence. Figure 3 shows the proportion of persistent HA users by HA fitting (bilateral/unilateral) and HL region. A detailed breakdown of persistence rates for each subcategory of HL together with patient counts is provided in Figure S1. Based on the regression results, Table 1 displays the statistical significance of the laterality main effect (indicated by star symbols as explained in the captions) as well as the ORs, Cis, and corresponding *p*-values of associations between laterality and long-term HA use persistence. Overall, the results show that persistence rates vary between about 36% and 68% across the various hearing regions and HA fitting categories.

**Table 1.** Regression Analyses of the Association Between Long-Term HA use Persistence and Laterality.

HA laterality main effects and pairwise comparisons (Odds Ratio [95% Confidence interval])				
Laterality	Region 1	Region 2	Region 3	Region 4
Total (main effect)	ns	***	***	***
Comparisons				
UB(US): BI	0.886 (0.782,1.004) ns	0.990 (0.762,1.286) ns	0.889 (0.785,1.008) ns	0.587 (0.549,0.628) ***
UD: BI	0.890 (0.759,1.045) ns			
UD: UB	1.005 (0.860,1.175) ns			
UW: BI		0.711 (0.654,0.774) ***	0.566 (0.513,0.624) ***	
UW: UB		0.719 (0.554,0.932) *	0.636 (0.545,0.743) ***	

This table shows statistical significance of the laterality main effect in the logistic regression models for each region as well as adjusted OR and 95% confidence intervals for each pairwise comparison together with statistical significance (***: *p* < .001; **: *p* < .01; *: *p* < .05; ns: *p* ≥ .05; UW excluded from modeling in region 1 due to low counts). BI = bilateral, UB/UW = unilateral better/worse ear; UD = unilateral without measurable PTA in contralateral ear, that is, single-sided deafness; US: unilateral with symmetric HL; PTA = pure-tone averages; HL = hearing loss; OR = odds ratio; HA = hearing aid.

In *region 1* (profound loss in WE, shaded blue in Figure 3), patients with worse-ear fittings were excluded from the regression analysis due to low counts. The statistical model did not find a statistical association between laterality and persistence, that is, there was no evidence of a difference in persistence between better-ear and bilateral HA fittings. Judging from the unadjusted persistence rates shown in Figure 3, this finding might be surprising because bilateral persistence appears to be appreciably higher than better-ear persistence. However, as a possible explanation Figure S1 indicates that the difference in persistence rates is driven by the severe-profound category where persistence as well as patient counts are particularly large for bilateral fittings. Within the other categories of region 1, persistence rates are much closer to each other. The two categories of unilateral better-ear fitting (i.e., without and with SSD) showed very similar levels of persistence.

In *region 2* (normal better-ear hearing; shown in turquoise in Figure 3), both better-ear and worse-ear fittings are included in the modeling. In this case, there is a highly significant association of persistence with fitting laterality. The pairwise comparisons between the individual fitting laterality categories reveal that this association is driven by low persistence rates in the worse-ear fitting group. ORs relative to bilateral and better-ear fittings are significantly lower than 1 (0.71 [95% CI: 0.65–0.77] and 0.72 [0.55–0.93], respectively) and unadjusted persistence is lower by about ten percentage points than in the other two groups. In contrast, and similar to the situation in region 1, there is no evidence of differences in persistence between bilateral and better-ear fittings. Their unadjusted persistence rates differ by only one percentage point.

The OR of 0.71 mentioned above means that the odds of being a persistent HA user are 29% lower for a patient with a unilateral worse-ear fitting relative to a patient who has a bilateral fitting, assuming that they agree with all other included characteristics. Odds are defined as p/(1−p) with *p* the probability of persistence.

Results for *region 3* (moderately asymmetric HL; orange in Figure 3) are comparable to those for region 2 with persistence for worse-ear fittings being significantly lower than for bilateral and better-ear fittings; the difference is around twenty percentage points. Note that about half of fittings in region 2 are worse-ear unilateral and their number is also appreciable in region 3 (see Figures 2 and S1). Persistence for better-ear fittings is only slightly lower than for bilateral fittings.

For symmetric HL (*region 4*; red in Figure 3), persistence rates for unilateral fittings are considerably smaller than for bilateral fittings (36% versus 57%, OR 0.59 [0.55–0.63]). Figure S1 shows that a large difference in persistence is found for all subclasses of symmetric HL.

### Analysis 2: Association of HA Laterality with Short-Term HA Usage and Total IOI-HA Score

There were 65,028 patients included in analysis 2.

### Analysis 2a: Association Between HA Fitting Laterality and Self-Reported Short-Term use

**Figure 4.**
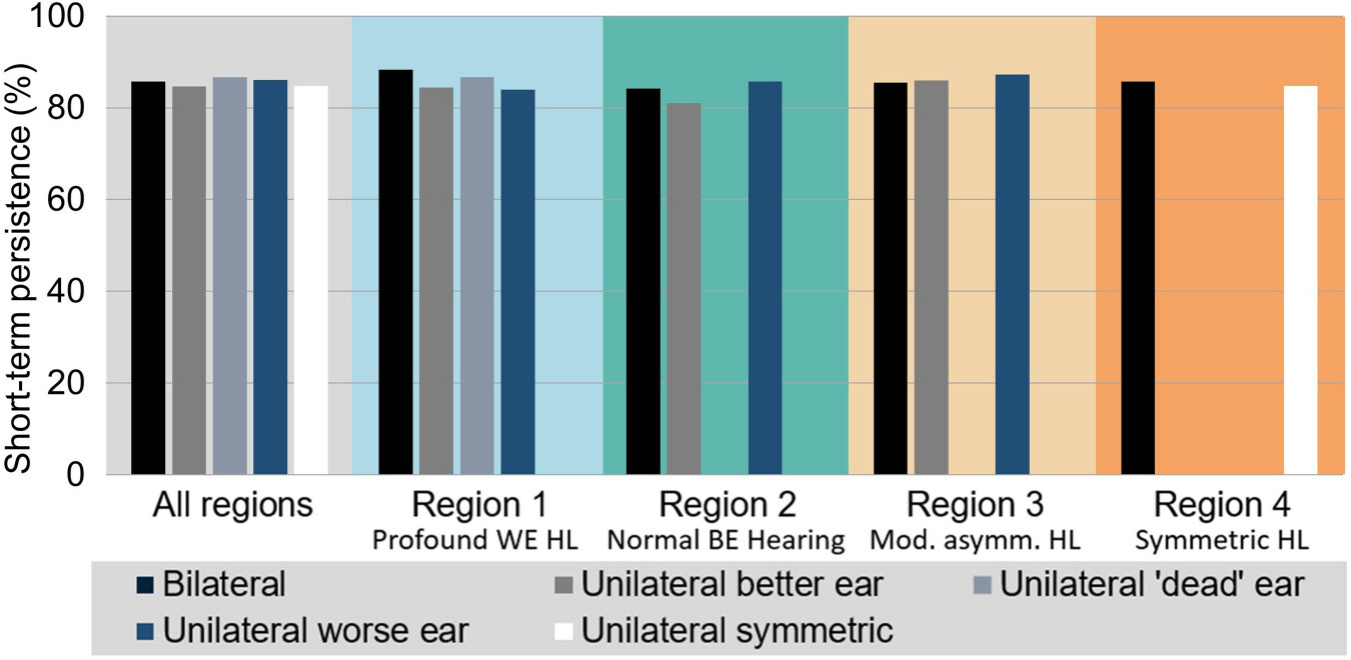
Short-term HA use persistence for the different types of HA laterality across the whole sample and separately for regions 1–4. Persistence rates refer to the proportion of patients who report using their HAs for at least 4 h per day. BE = better ear, WE = worse ear; HA = hearing aid; HL = hearing loss.

The results for the comparisons of short-term HA use as measured by IOI-HA item 1 are presented in Figure 4, Figure S2, and Table 2. Self-reported short-term usage rates (i.e., proportion of patients wearing their HAs for at least 4 h/day) range from 81% to 88%. In contrast to the results for long-term persistence, the statistical comparisons do not reveal any significant differences in usage between fitting laterality categories (note that in region 3, that is, moderately asymmetric HL, the comparison between worse-ear and bilateral fittings finds a significant difference but there is no significant main effect). In other words, there is no evidence that short-term usage varies among fitting laterality categories.

**Table 2.** Regression Analyses of the Association Between Short-Term HA Usage and Laterality.

HA laterality main effects and pairwise comparisons (Odds ratio [95% Confidence interval])				
Laterality	Region 1	Region 2	Region 3	Region 4
Total (Main effect)	ns	ns	ns	ns
Comparisons				
UB(US): BI	0.903 (0.654,1.246) ns	0.735 (0.378,1.428) ns	1.015 (0.728,1.414) ns	0.951 (0.803,1.125) ns
UD: BI	1.134 (0.738,1.743) ns			
UD: UB	1.256 (0.828,1.905) ns			
UW: BI		1.127 (0.890,1.427) ns	1.309 (1.000,1.713) *	
UW: UB		1.534 (0.792,2.971) ns	1.290 (0.850,1.958) ns	

Table layout as for Table 1. BI = bilateral; UB/UW = unilateral better/worse ear; UD = unilateral without measurable PTA in contralateral ear, that is, single-sided deafness; US = unilateral with symmetric HL; PTA = pure-tone averages; HL = hearing loss; HA = hearing aid.

### Analysis 2b: Association Between HA Laterality and Total IOI-HA Score

**Figure 5.**
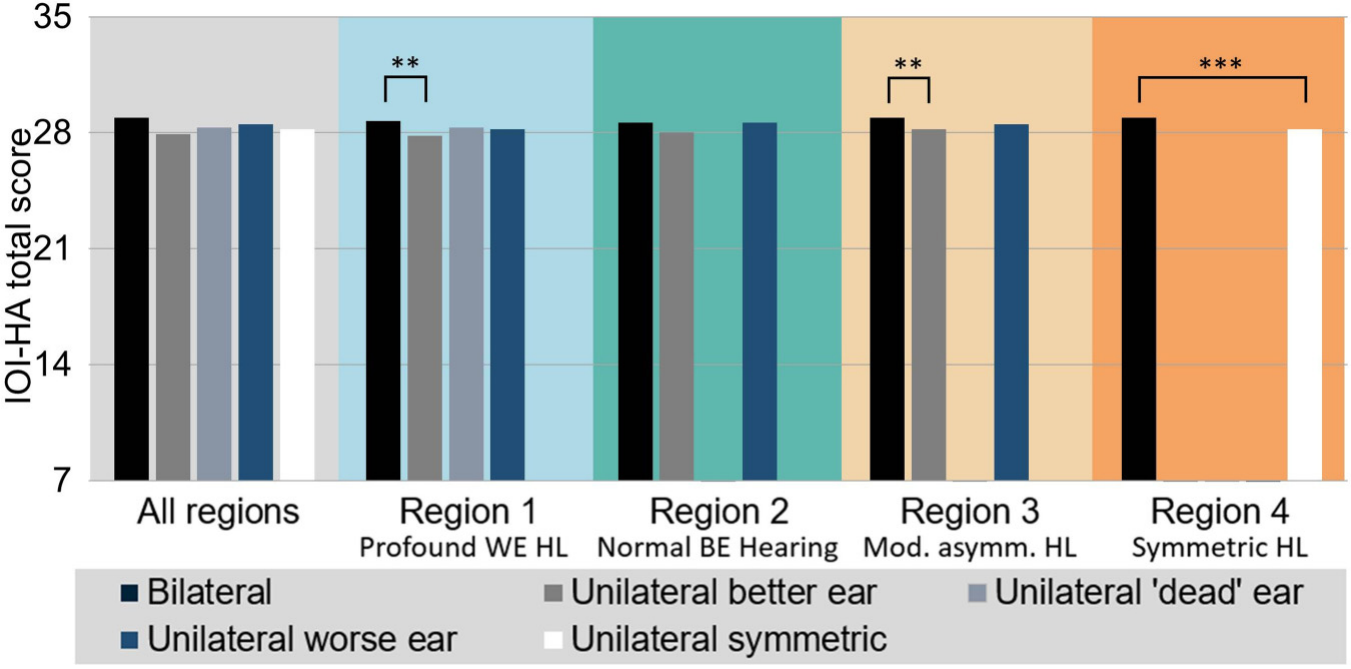
IOI-HA total score for the different types of HA laterality across the whole sample and separately for regions 1–4. Statistically significant comparisons identified by the linear regression models are highlighted (see Table 3). The limits on the vertical axis cover the full range of possible total scores. BE = better ear, WE = worse ear; IOI-HA = International Outcome Inventory for Hearing Aids; HL = hearing loss.

Analyses of the association between HA fitting laterality and total IOI-HA scores are summarized in Figure 5, Figure S3, and Table 3. Across regions and fitting laterality categories, average total IOI-HA scores range from 27.8 to 28.9. The adjusted differences in IOI-HA scores between laterality types shown in Table 3 are obtained from separate multiple linear regression models for each of the four regions. The regression models find significant main effects of laterality of fitting in regions 1 (profound WE HL), 3 (moderately asymmetric HL), and 4 (symmetric HL). Pairwise comparisons between fitting laterality categories reveal that the main effects are driven by better-ear fittings and unilateral fittings for symmetric HL, which have significantly lower average total scores than bilateral fittings (adjusted differences between 0.61 and 0.72 points, respectively). The difference in region 2 (normal BE hearing) is of comparable magnitude (0.65 points) but the comparison is not statistically significant, presumably due to low patient counts for better-ear fittings. There are no significant differences for any of the other pairwise comparisons in the four regions.

**Table 3.** Regression Analyses of the Association Between Total IOI-HA Score and Laterality.

HA laterality main effects and pairwise comparisons (Beta coefficient [95% Confidence interval])				
Laterality	Region 1	Region 2	Region 3	Region 4
Total (main effect)	**	ns	**	***
Comparisons				
UB(US): BI	−0.722 (−1.195, −0.250) **	−0.654 (−1.677,0.369) ns	−0.689 (−1.143,−0.234) **	−0.605 (−0.841,−0.370) ***
UD: BI	−0.239 (−0.857,0.380) ns			
UD: UB	0.483 (−0.129,1.096) ns			
UW: BI		−0.098 (−0.436,0.241) ns	−0.314 (−0.671,0.044) ns	
UW: UB		0.556 (−0.459,1.572) ns	0.375 (−0.188,0.938) ns	

This table shows the statistical significance of the laterality main effect in the linear regression models for each region as well as the score difference (beta coefficient) for each pairwise comparison between laterality types after adjusting for covariates. BI = bilateral; UB/UW = unilateral better/worse ear; UD = unilateral without measurable PTA in contralateral ear, that is, single-sided deafness; US = unilateral with symmetric HL; IOI-HA = International Outcome Inventory for Hearing Aids; PTA = pure-tone averages; HL = hearing loss; HA = hearing aid.

To facilitate the interpretation of the adjusted differences (beta coefficients) shown in Table 3 we consider the value of −0.722 for better-ear versus bilateral fittings in region 1. This value means that the total IOI-HA score for a patient with a unilateral better-ear fitting is expected to be lower by about 0.72 points relative to a bilateral patient who otherwise agrees on all characteristics used in the statistical modeling.

Note that, for brevity, the present paper does not discuss the associations between our outcome measures (i.e., HA long-term and short-term persistence, total IOI-HA score) and the covariates used in the regression models. These will be described thoroughly in future publications. However, as comparisons show, there is a clear correspondence between the patterns in the unadjusted statistics and the adjusted ORs and B coefficients obtained from the regressions. This implies that the association between outcomes and laterality is not strongly affected by covariate effects. In fact, the ORs and B coefficients presented here are very similar to the estimates obtained in models without covariates (results not shown). For the present purposes, the covariates can therefore be considered nuisance variables that mainly serve to explain some of the variations in the outcomes.

### Summary

Our key finding is that long-term HA use persistence is lower for worse-ear HA fittings and unilateral fittings for symmetric HL compared to bilateral and better-ear fittings. In contrast, there was no difference in self-reported short-term HA usage (IOI-HA Q1) across laterality categories. However, the total IOI-HA score was poorer for better-ear fittings and unilateral fittings for symmetric HL compared to bilateral fittings.

## Discussion

In our sample of US Veterans who were first-time recipients of HAs in VHA audiology between 2012 and 2014, the vast majority of fittings were bilateral (about 93% among the patients in Figure 2). However, the proportion of unilateral fittings depended strongly on patients’ binaural HL pattern and the degree of hearing asymmetry, varying between 84% of patients with normal hearing in the BE and profound HL in the worse (i.e., extreme asymmetry) and 0.4% for patients with moderate symmetric HL (Figure 2). Furthermore, at a given level of asymmetry, unilateral fittings became more common as hearing levels approached the end of the testing ranges (e.g., 14.5% and 4.4% for symmetric normal hearing and profound symmetric HL, respectively, compared to 0.4% for moderate symmetric HL).

The overall proportion of unilateral fittings in our patient group (about 7%) is lower than those reported by others. For example, Boymans et al. (2009) reported that 41% of fittings were unilateral among their representative sample of 1,000 HA recipients in the Netherlands, while Arlinger et al. (2017) reported a rate of 19% in their sample covering half of all HA fittings in Sweden between 2012 and 2016. We do not know what is driving the low proportion of unilateral fittings in our sample. Cost has been proposed as a factor in the selection of unilateral fittings (Dillon, 2012; Schilder et al., 2017), and as VHA HAs are provided free of charge to patients, the removal of this barrier might play a role here. However, our results are also not easily reconciled with previous studies on patient preference. Specifically, Cox et al. (2011) found that 46% of their sample of 94 participants preferred to use one HA, while Glyde et al. (2021) reported that 13% of their 68 participants preferred a unilateral device, with 9% having no preference.

Our observation that unilateral fittings became more common with increased hearing asymmetry is in line with the findings of Boymans and Dreschler (2011). They reported that the average hearing asymmetry for unilateral fittings was 22 dB, while for bilateral fittings it was 8 dB. In 65% of unilateral fittings with an asymmetry of at least 10 dB, the user received a better-ear fitting. In our sample, worse-ear fittings were more common than better-ear fittings overall, but the relative proportions of each were dependent on the HL pattern. More specifically, worse-ear fittings were more common when the unfitted ear had normal hearing and the fitted ear had mild to severe HL. Better-ear fittings, on the other hand, were predominant when the unfitted ear had a profound loss and the fitted ear had normal hearing (according to the PTA) to moderate HL. Patients with SSD also commonly received unilateral fittings especially if the HL in the BE was mild to moderate. Bilateral fittings for patients with SSD were uncommon, as were unilateral fittings for patients with symmetric HL—who by far constitute the largest patient group (79%).

Altogether, these observations are in line with the intuitive expectation that unilateral fittings make most sense when the patient has one ear with normal hearing (so there is no *need* for a HA in this ear) or when one ear has profound HL (so there is little or no *benefit* to fitting this ear). We are currently not aware of any comparable reports in the literature on distributions of unilateral HA fittings between BE and WE.

As noted above, we cannot know what factors influenced the choice of fitting type. In particular, it is unclear why in regions 1 (profound worse-ear HL) and 2 (normal better-ear hearing) some patients are fitted unilaterally and some bilaterally. However, further analysis suggested that the HL configuration plays a role. In region 1, excluding all SSD patients, better-ear fittings tend to be associated with more HL in the unfitted ear than bilateral fittings. In region 2, bilateral fittings appear to co-occur with more pronounced *high-frequency* HL in the BE. Specifically, average 8 kHz thresholds are higher by 11–14 dB compared to worse-ear fittings across the three HL patterns in this region. We considered the possibility that a second HA was provided to patients in region 2 to help with tinnitus in that ear (Henry et al., 2015; Suzuki et al., 2021), but the prevalence of tinnitus (as determined by the presence of a diagnostic code) among bilateral fittings was the same as that for worse-ear fittings. However, in analyses covering the whole sample, we found other infrequently encountered pathologies, such as ear infection or stroke, to be associated with an increased likelihood of unilateral fittings. This may indicate that the presence of certain comorbidities influences decision-making.

Perhaps our most striking results are those showing substantially lower HA use persistence for worse-ear fittings and unilateral fittings under symmetric HL compared to bilateral and better-ear fittings, with differences in unadjusted persistence rates ranging from 9 to 29 percentage points across the four regions of binaural HL configurations. Correspondingly, adjusted ORs are between 0.57 and 0.72. Unadjusted persistence rates for better-ear fittings are typically within a few percentage points of those for bilateral fittings. Adjusted ORs (ranging from 0.89 to 0.99) thus do not show evidence of differences in persistence between these two fitting laterality types. Persistence rates for unilateral fittings with single-side deafness in region 1 (profound worse-ear HL) are very close to those for better-ear fittings.

Results consistent with ours were reported by Bisgaard and Ruf (2017) who found higher daily use for bilateral than unilateral fittings (9.1 h versus 7.8 h, respectively) and fewer bilateral than unilateral users having their HAs “in the drawer” (i.e., no daily use time)—3.9% versus 9.4%, respectively. A representative survey of HA owners in Switzerland (Bertoli et al., 2010) found that among patients with symmetric HL, unilateral fitting was significantly associated with non-regular HA use (*N* = 6027, OR = 1.38 relative to bilateral). In this study, use was measured through self-report and non-regular use defined as HAs being worn for less than 1 day per week.

We observed no association of the dichotomized Q1 IOI-HA score (short-term HA usage) with HA fitting laterality, and total IOI-HA score showed only that better-ear fittings and unilateral fittings for symmetric HL scored lower than bilateral fittings. In contrast, other studies have found stronger effects in favor of bilateral fittings reflected in IOI-HA scores. For instance, Arlinger et al. (2017) reported that bilateral users had significantly higher daily HA use than unilateral users (mean Q1 IOI-HA score 4.14 versus 3.95) as well as having IOI-HA total scores higher by 1.12 points. Wang et al. (2022) reported similar findings, with bilateral users having median IOI-HA total score 2 points higher than unilateral users. These differences between bilateral and unilateral fittings are considerably larger than were seen in our data (0.6–0.7 points in favor of bilateral fittings). Self-reported short-term outcome measured by the IOI-HA (Q1 and total score) was less sensitive to differences in laterality of fitting than our HA use persistence measure. Explanations for this might lie in the fact that patients self-select whether to return an IOI-HA measure, whereas the battery ordering data is collected for all patients. However, we cannot determine what precise mechanism(s) might be acting to introduce bias or variance in the IOI-HA scores.

Our results are not easily reconciled with the findings of Swan et al. (1986, 1987) who reported a clear patient preference for the WE in unilateral fittings. It was hypothesized that this choice is based on a desire to minimize the disability in the most disadvantageous listening situations (Swan & Gatehouse, 1987a, 1987b). In contrast, the present study shows substantially better long-term persistence for better-ear fittings for HL configurations in regions 2 (normal better-ear hearing), and 3 (moderately asymmetric HL), and no statistically significant differences in self-reported short-term outcomes. While we cannot provide an explanation for this discrepancy, we note that our results suggest that initial patient preference may not be the only determinant of long-term HA outcomes. It is possible that other effects turn out to be more impactful in the long run; in fact, in their study of outcomes of HA use in patients with worse-ear fittings, Noh and Lee (2023) note that they “have often encountered cases in which the side fitted with a HA changed from the WE to the BE during the process of counselling and fitting.” Swan and Gatehouse (1987a, 1987b) report a higher benefit of better-ear fittings in certain listening situations, and recent results by Smeds et al. (2015) suggest that HA users spend little time in the most difficult listening situations. Therefore, they may ultimately do better with a better-ear fitting. It should also be noted that HA technology has advanced considerably between the time of the studies of Swan et al. (early to mid 1980s) and the current work (2012–2014). These advances imply that the findings of Swan et al. may not be directly applicable to the patients in our study.

### Clinical Implications

While the analyses do not allow us to claim a causal effect of HA fitting laterality on HA use persistence, we do show substantially worse long-term outcomes for worse-ear fittings and unilateral fittings under symmetric HL. Bilateral and better-ear fittings have similar long-term outcomes, but short-term outcomes being poorer for better-ear than bilateral fittings. Our findings thus provide strong empirical evidence for the recommendation made by others (e.g., Boymans & Dreschler, 2011; Dillon, 2012; Glyde et al., 2021; Kiessling et al., 2006; Noble & Gatehouse, 2006) that HAs should be fitted bilaterally under almost all circumstances. We also note that HAs today can offer even more advanced technological features compared to what is available with the HAs provided to our sample of patients. Specifically, left and right HAs can now be paired to enable binaural digital sound processing, which might further enhance the value of bilateral fittings over unilateral ones. However, if a unilateral fitting is chosen, our data suggest that the BE should be fitted. We emphasize that this recommendation is based solely on long-term HA use persistence. Other factors, such as sound quality, convenience, etc. will also affect the decision of which ear to fit (Dillon, 2012; Glyde et al., 2021). Ultimately, as stated by Cox et al. (2011), “a patient's decision about his or her own best treatment must be respected” (p. 195).

### Limitations

Our study has several limitations that warrant mention. First, our sample consists of (mostly male) US Veterans who received audiological care through the VHA system. Therefore, it is unclear how generalizable our results are to other patient groups and models of care. However, given the size and diversity of our sample, as well as the wide range of audiological care providers within the VHA system we are confident that our results and conclusions remain relevant within a broader context, especially because the VA healthcare system is somewhat analogous to some nationalized healthcare systems.

Second, our persistence measure is calculated using battery order data (Zobay et al., 2021). Even though battery supplies distributed by the VA are calibrated to 6-month full-time usage of HAs, it is probable that this calibration is imperfect which would affect our measure of persistence. To further examine this, we repeated our analyses using persistence at 3 years after fitting (instead of 2) by extending the time window for battery orders to 30 months. This should minimize the effects of differing battery consumption. As can be seen in Table S1 and Figure S4 of the supplementary material, the analysis yields patterns of association between persistence and laterality very similar to those found for 2-year persistence (Table 1 and Figure S1). In other words, it is unlikely that our results are significantly skewed by inaccurate calibration of HA orders. On a related note, we cannot determine whether some patients who received a bilateral fitting in fact used only one of their HAs. If this were the case their battery orders would last about twice as long as expected. Therefore, these patients would likely be classified as non-persistent due to the reduced frequency of battery orders. The “true” persistence rates for patients with bilateral fittings would thus be higher than the reported estimates.

In general, it is unavoidable that our persistence measure has limited sensitivity and specificity, that is, some patients whom we classify as non-persistent are in fact persistent and vice versa. However, these limitations will act to attenuate any associations between the outcome and the predictor variables. It is therefore likely that we would see even stronger relationships if we could use a “perfect” persistence measure in our modeling.

Third, patients for whom we have IOI-HA data are not a random subsample of the full set of patients. An informal survey of VA audiologists indicated that IOI-HA questionnaires are often administered at a HA follow-up appointment. Patients who stop using their HAs prior to a follow-up visit will be less represented, as would patients who choose not to attend a follow-up appointment. Some of this latter group might be highly successful users, while others might be unmotivated users. Unfortunately, we have no way to obtain data regarding why patients did not complete an IOI-HA. However, a comparison of HA use persistence for patients with and without IOI-HA data (Supplemental Figures S2 and S3) shows persistence to be lower by about 10 percentage points for patients without IOI-HA data relative to those with IOI-HA data. Nonetheless, the laterality of fittings does not differ substantially between patients with and without IOI-HA data (Supplemental Figures S4 and S5), and we, therefore, consider comparisons of persistence within the IOI-HA subsample to be valid.

Fourth, as noted above, HA technology has made further advances over the past few years, and we expect that the advantages of bilateral fittings will be even more pronounced with current devices.

Finally, we note that our data do not allow us to make assertions of causal effects of HA laterality on HA use persistence. It is possible that some patients choose a single device because they expect that one HA will be easier to manage than two. A patient looking for ease of use might also be more likely to stop using their device than someone who is prioritizing their hearing ability. However, many studies have demonstrated the advantages of bilateral fittings (e.g., Boymans & Dreschler, 2011; Dillon, 2012; Noble & Gatehouse, 2006;) and it is therefore plausible that a higher degree of satisfaction and clearer benefits will lead to more persistent HA use. In addition, the observation that persistence rates for better-ear fittings were significantly higher than those for worse-ear fittings points toward a causal influence of laterality on long-term HA usage.

In spite of these limitations and uncertainties, we conclude that our results indicate that bilateral aids yield the best short- and long-term outcomes, and while unilateral devices can result in similar outcomes on some measures, we did not identify any HL configuration for which a bilateral fitting would lead to a poorer outcome than a unilateral fitting. However, if there is a reason to fit a unilateral HA, our results provide empirical evidence in favor of fitting the BE rather than the worse one.

## Supplemental Material

sj-docx-1-tia-10.1177_23312165231195987 - Supplemental material for Fitting a Hearing Aid on the Better Ear, Worse Ear, or Both: Associations of Hearing-aid Fitting Laterality with Outcomes in a Large Sample of US Veterans Click here for additional data file. Supplemental material, sj-docx-1-tia-10.1177_23312165231195987 for Fitting a Hearing Aid on the Better Ear, Worse Ear, or Both: Associations of Hearing-aid Fitting Laterality with Outcomes in a Large Sample of US Veterans by Oliver Zobay, Graham Naylor, Gabrielle H. Saunders and Lauren K. Dillard in Trends in Hearing
