# Supplementary material for: Fitting a Hearing Aid on the Better Ear, Worse Ear, or Both: Associations of Hearing-aid Fitting Laterality with Outcomes in a Large Sample of US Veterans
Source: Trends Hear. 2023 Aug 24;27:23312165231195987. doi: 10.1177/23312165231195987 (PMC10467180; doi:10.1177/23312165231195987)
Supplement: sj-docx-1-tia-10.1177_23312165231195987 - Supplemental material for Fitting a Hearing Aid on the Better Ear, Worse Ear, or Both: Associations of Hearing-aid Fitting Laterality with Outcomes in a Large Sample of US Veterans [file sj-docx-1-tia-10.1177_23312165231195987.docx]

**Supplemental material**

**Contents:**

1. Covariates used in the logistic and linear regression models of analyses 1, 2a and 2b (p. 2)
2. Figures S1-S3 (p. 3-4): Long-term and short-term HA use persistence rates and total IOI-HA score by degree of better-ear and worse-ear hearing loss. These figures provide additional, more detailed data related to analyses 1, 2a and 2b.
3. Figure S4 and Table S1 (p. 5): Long-term HA use persistence rates with persistence assessed at 3 years after fitting and with 2.5-year time window for battery order
4. Figures S5 and S6 (p. 6): Long-term HA use persistence rates for patients with and without IOI submission by degree of better-ear and worse-ear hearing loss
5. Figures S7 and S8 (p. 7): Distributions of laterality types for patients with and with IOI submission by degree of better-ear and worse-ear hearing loss

*Covariates for logistic and linear regression models*

Variables that were found to be associated with HA use persistence and IOI-HA outcomes were used as covariates in our statistical models. Specifically, we included:

- Age at HA fitting: Included as continuous variable in second order to model a quadratic dependence.
- HA type (3 categories): behind the ear (BTE), in the ear (ITE), receiver in the canal (RIC).
- Marital status at a time as close as possible to the HA fitting (5 categories): Divorced, Married, Never Married, Separated, Widowed.
- Race (5 categories): American Indian or Alaska Native, Asian, Black or African American, Native Hawaiian or other Pacific Islander, White (including White not of Hispanic origin)
- Ethnicity (2 categories): Hispanic or Latino, not Hispanic or Latino
- Income (5 categories): the median income of the zip code area of the patient’s address at the time of the HA fitting, based on data from the American Community Survey (ACS) for the 5-year period from 2015-2019 available at data.census.gov. In the absence of more detailed information, this variable served as a proxy for socio-economic status. The data were discretized to five categories by splitting at quintiles.
- Urban/rural status (4 categories): Urban, Large Town, Small Town, Rural. Categories were assigned using the rural-urban commuting area (RUCA) codes pertaining to patients’ zip codes (Hart et al. 2005; see also [Rural Urban Commuting Area Codes Data (washington.edu),](https://depts.washington.edu/uwruca/ruca-uses.php) Categorization A).
- Short-term hearing care (continuous variable): the number of hearing-care outpatient appointments in the VA system within the first 6 months of the HA fitting. This covariate was not included in models for Analysis 2 (see below), in which outcomes of interest were measured over a six-month period after HA fitting.
- Multimorbidity index (continuous variable): based on the Chronic Condition Indicator (Healthcare Cost and Utilization Project 2016) and determined as the number of body systems for which the patient has at least one chronic condition (excluding hearing loss): range: 0-18 (see Saunders et al., 2021 for details).

Hart, L. G., Larson, E. H., & Lishner, D. M. (2005). Rural definitions for health policy and research. *American Journal of Public Health*, *95*(7), 1149-1155. <https://doi.org/10.2105/AJPH.2004.042432>

Healthcare Cost and Utilization Project. (2016). *Agency for Healthcare Research and Quality.* Rockville, MD. <www.hcup-us.ahrq.gov/toolssoftware/chronic/chronic.jsp>.

Saunders, G. H., Dillard, L. K., Zobay, O., Cannon, J. B., & Naylor, G. (2021). Electronic Health Records As a Platform for Audiological Research: Data Validity, Patient Characteristics, and Hearing-Aid Use Persistence Among 731,213 U.S. Veterans. *Ear & Hearing*, *42*(4), 927-940. [https://doi.org/10.1097/AUD.0000000000000980](https://doi.org/10.1097/AUD.0000000000000980%20)


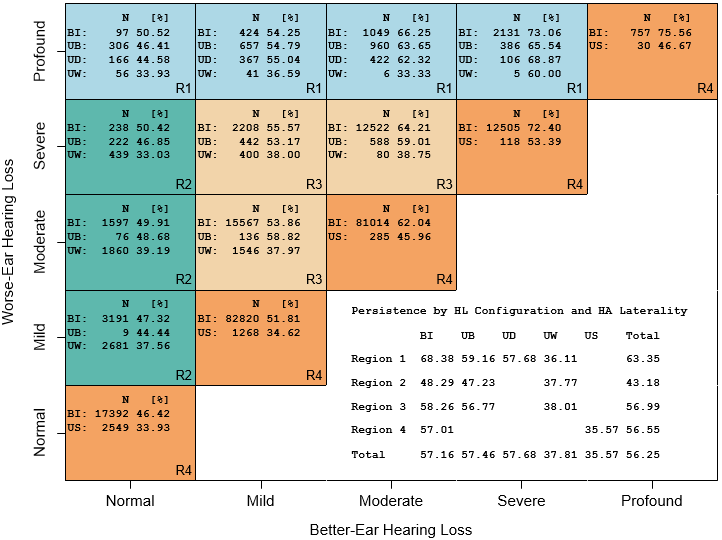


Figure S1. Patient counts and unadjusted long-term HA use persistence rates for new users by laterality category and better-ear and worse-ear hearing loss. The table in the bottom right corner shows persistence rates for the four regions and overall. HA fitting categories BI: bilateral, UB/UW: unilateral better/worse ear, UD: unilateral without measurable PTA in contralateral ear, i.e., single-sided deafness, US: unilateral with symmetric HL.


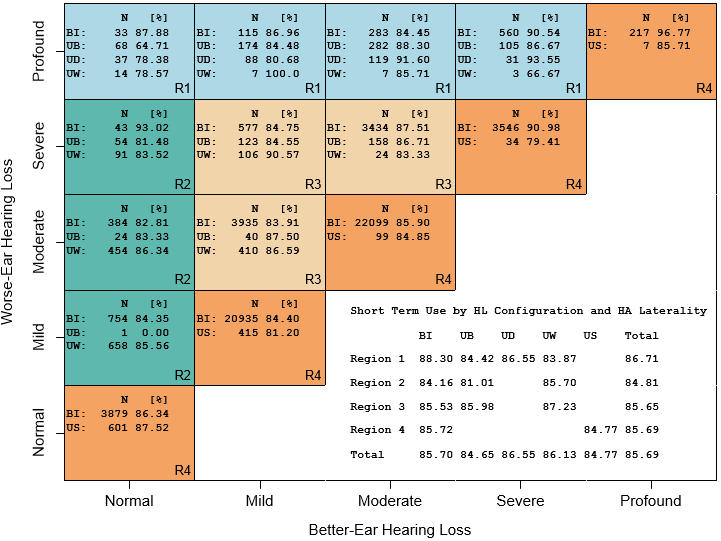


Figure S2. Patient counts and unadjusted short-term HA usage for new users by laterality category and better-ear and worse-ear hearing loss. The table in the bottom right corner shows usage rates for the four regions and overall. HA fitting categories BI: bilateral, UB/UW: unilateral better/worse ear, UD: unilateral without measurable PTA in contralateral ear, i.e., single-sided deafness, US: unilateral with symmetric HL.


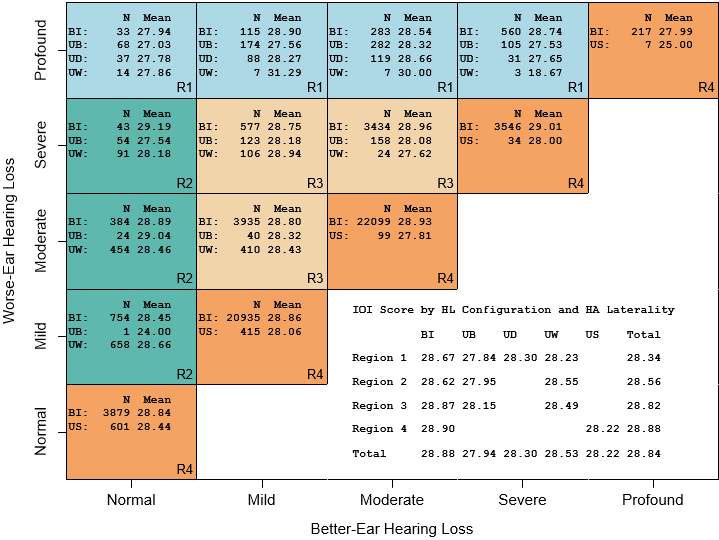


Figure S3. Patient counts and unadjusted average total IOI-HA scores for new users by laterality category and better-ear and worse-ear hearing loss. The table in the bottom right corner shows average total scores for the four regions and overall. HA fitting categories BI: bilateral, UB/UW: unilateral better/worse ear, UD: unilateral without measurable PTA in contralateral ear, i.e., single-sided deafness, US: unilateral with symmetric HL.


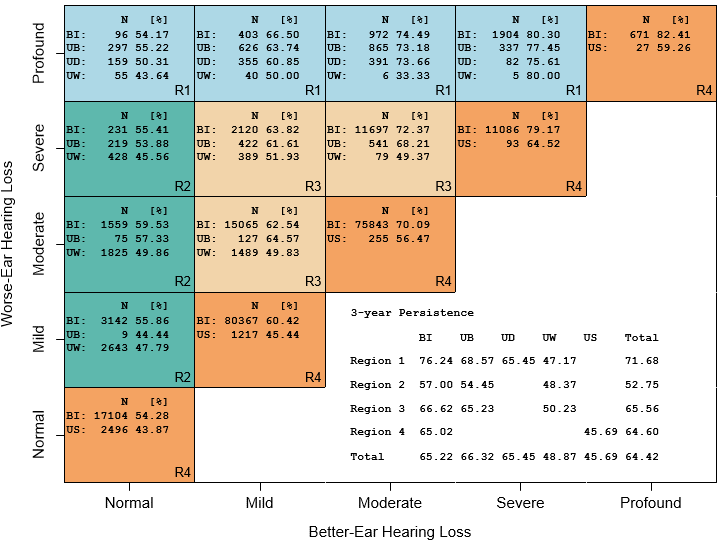


Figure S4. Long-term HA use persistence rates with persistence assessed at 3 years after fitting and with 2.5-year time window for battery order. HA fitting categories BI: bilateral, UB/UW: unilateral better/worse ear, UD: unilateral without measurable PTA in contralateral ear, i.e., single-sided deafness, US: unilateral with symmetric HL.

Table S1. Regression analyses of the association between laterality and HA use persistence assessed at 3 years after fitting and with 2.5-year time window for battery order. The table shows statistical significance of the laterality main effect in the logistic regression models for each region as well as adjusted odds ratio (OR) and 95% confidence intervals for each pairwise comparison together with statistical significance (***: p<0.001; **: p<0.01; *: p<0.05; ns: p≥0.05; UW excluded from modelling in region 1 due to low counts). HA fitting categories BI: bilateral, UB/UW: unilateral better/worse ear, UD: unilateral without measurable PTA in contralateral ear, i.e., single-sided deafness, US: unilateral with symmetric HL.

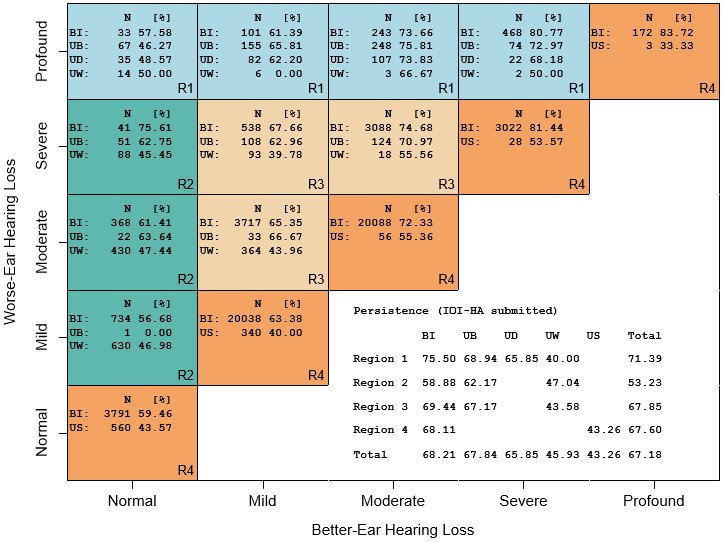


Figure S5. Long-term HA use persistence rates for patients *with* IOI submission. HA fitting categories BI: bilateral, UB/UW: unilateral better/worse ear, UD: unilateral without measurable PTA in contralateral ear, i.e., single-sided deafness, US: unilateral with symmetric HL.


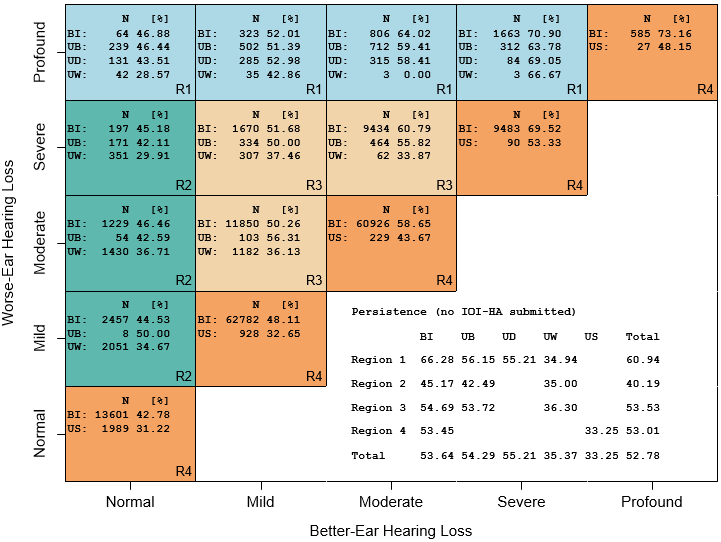


Figure S6. Long-term HA use persistence rates for patients *without* IOI submission. Counts in this diagram and in Figure S5 add up to those in Figure S1. HA fitting categories BI: bilateral, UB/UW: unilateral better/worse ear, UD: unilateral without measurable PTA in contralateral ear, i.e., single-sided deafness, US: unilateral with symmetric HL.


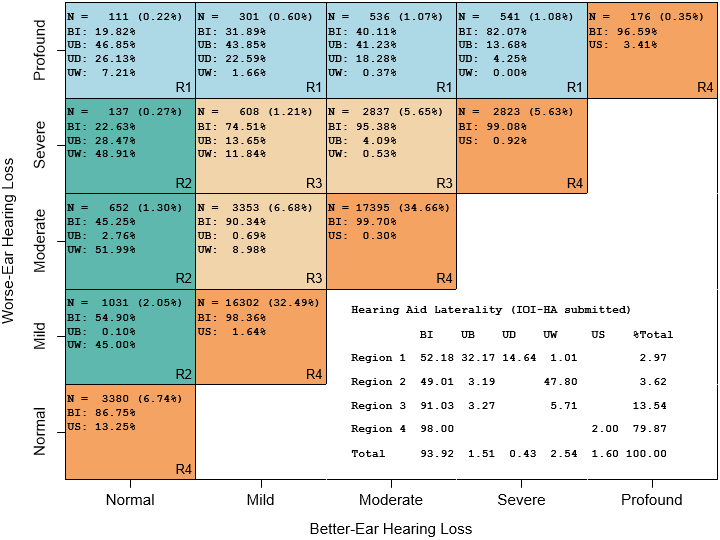


Figure S7. Distributions of laterality types for patients *with* IOI submission. HA fitting categories BI: bilateral, UB/UW: unilateral better/worse ear, UD: unilateral without measurable PTA in contralateral ear, i.e., single-sided deafness, US: unilateral with symmetric HL.


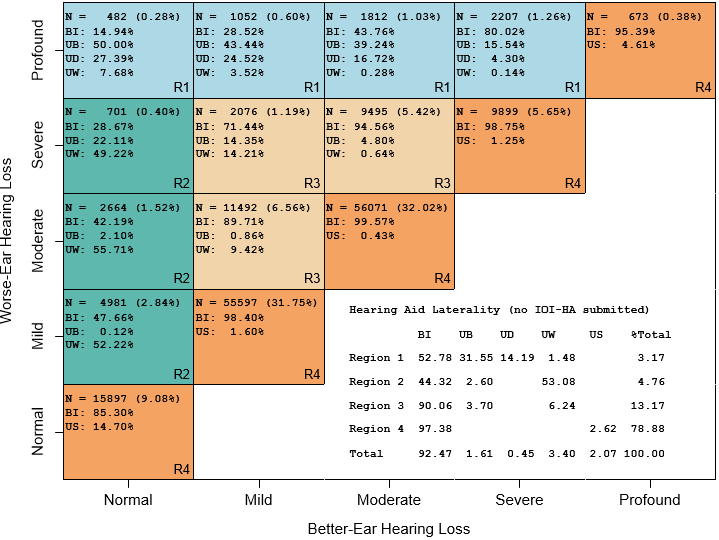


Figure S8. Distributions of laterality types for patients *without* IOI submission. HA fitting categories BI: bilateral, UB/UW: unilateral better/worse ear, UD: unilateral without measurable PTA in contralateral ear, i.e., single-sided deafness, US: unilateral with symmetric HL.
